# Degradation of epigallocatechin and epicatechin gallates by a novel tannase Tan_Hcw_ from *Herbaspirillum camelliae*

**DOI:** 10.1186/s12934-021-01685-1

**Published:** 2021-10-12

**Authors:** Jia Lei, Yong Zhang, Xuechen Ni, Xuejing Yu, Xingguo Wang

**Affiliations:** 1 State Key Laboratory of Biocatalysis and Enzyme Engineering, Hubei Collaborative Innovation Center for Green Transformation of Bio-Resources, Hubei Key Laboratory of Industrial Biotechnology, School of Life Sciences, Hubei University, Wuhan, 430062 China; 2 Xianning Central Hospital, Tongji Xianning Hospital, Xianning, Hubei Province China

**Keywords:** *Herbaspirillum*, Tannase, Enzymatic characterizations, Kinetic parameters, Secretary proteins

## Abstract

**Background:**

*Herbaspirillum camelliae* is a gram-negative endophyte isolated from the tea plant. Both strains WT00C and WT00F were found to hydrolyze epigallocatechin-3-gallate (EGCG) and epicatechin-3-gallate (ECG) to release gallic acid (GA) and display tannase activity. However, no tannase gene was annotated in the genome of *H. camelliae* WT00C.

**Results:**

The 39 kDa protein, annotated as the prolyl oligopeptidase in the NCBI database, was finally identified as a novel tannase. Its gene was cloned, and the enzyme was expressed in *E. coli* and purified to homogeneity. Moreover, enzymatic characterizations of this novel tannase named Tan_Hcw_ were studied. Tan_Hcw_ was a secretary enzyme with a Sec/SPI signal peptide of 48 amino acids at the N-terminus, and it catalyzed the degradation of tannin, methyl gallate (MG), epigallocatechin-3-gallate (EGCG) and epicatechin-3-gallate (ECG). The optimal temperature and pH of Tan_Hcw_ activities were 30 °C, pH 6.0 for MG and 40 °C, pH 7.0 for both EGCG and ECG. Na^+^, K^+^ Mn^2+^ and Triton-X100, Tween80 increased the enzyme activity of Tan_Hcw_, whereas Zn^2+^, Mg^2+^, Hg^2+^, EMSO, EDTA and β-mercaptoethanol inhibited enzyme activity. *K*_*m*_*, k*_*cat*_ and *k*_*cat*_* /K*_*m*_ of Tan_Hcw_ were 0.30 mM, 37.84 s^−1^, 130.67 mM^−1^ s^−1^ for EGCG, 0.33 mM, 34.59 s^−1^, 105.01 mM^−1^ s^−1^ for ECG and 0.82 mM, 14.64 s^−1^, 18.17 mM^−1^ s^−1^ for MG, respectively.

**Conclusion:**

A novel tannase Tan_Hcw_ from *H. camelliae* has been identified and characterized. The biological properties of Tan_Hcw_ suggest that it plays a crucial role in the specific colonization of *H. camelliae* in tea plants. Discovery of the tannase Tan_Hcw_ in this study gives us a reasonable explanation for the host specificity of *H. camelliae*. In addition, studying the characteristics of this enzyme offers the possibility of further defining its potential in industrial application.

**Supplementary Information:**

The online version contains supplementary material available at 10.1186/s12934-021-01685-1.

## Background

Tannase, also known as tannin acyl-hydrolase [EC 3.1.1.20], catalyzes the hydrolysis of ester bonds in gallotanins, epigallocatechin gallate, epicatechin gallate, and gallic acid esters. The hydrolysis of tannic acids by tannase releases gallic acid, some galloyl esters, and glucose [1–3]. Tannases have wide application in food, feed, beverage, chemical and pharmaceutical industries. However, their enzyme reagents came mainly from microbial cells or crude cellular extracts without enzyme isolation. Thus, little is known about the tannases at the molecular level because most tannase coding genes have not been cloned and tested, which are indeed worthy of exploration. Previous studies have reported that tannases could be obtained from various sources, for instance, microbial, fungi, vegetal and animal [1, 3]. Analysis of tannase genes from the database has demonstrated that the amino acid sequences of tannases are rather divergent. Nevertheless, a distinct active site motif (Gly-X-Ser-X-Gly) has been pinpointed by analyzing amino acid sequences of bacteria, yeast, and fungal tannases [3, 4]. Rivas et al. (2019) have classified tannases into two categories: tannases and feruloyl esterases/tannases. The former includes subtype A tannases (absence of catalytic Asp) and subtype B tannases (with catalytic Asp), while the latter consists of “CS-D-HC” feruloyl esterase/tannase and non- “CS-D-HC” feruloyl esterase/tannase [3].

In our previous study, *herbaspirillum camelliae* WT00C and WT00F were isolated from the tea plant (*Camellia sinensis* L) and classified as a novel species in the *Herbaspirillum* genus [5, 6]. As a gram-negative endophyte, *H. camelliae* WT00C and WT00F entered the tea plant via vulnus and colonized only in the stem and old leaves of the tea plant [7]. Although *H. camelliae* WT00C and WT00F were unable to fix nitrogen, both strains not only stimulated tea-plant growth and development but also reduced selenate to form elemental selenium (Se^0^) and enhanced selenium enrichment in tea [7–9]. The genome of *H. camelliae* WT00C was sequenced and deposited in the GenBank database (Acc#: KV880769.1) [10]. In the recent study, TLC and HPLC analysis found that *H. camelliae* WT00C and WT00F effectively degraded EGCG (epigallocatechin-3-gallate) and ECG (epicatechin-3-gallate) to release GA (gallic acid). However, they did not hydrolyze EGC (epigallocatechin), EC (epicatechin), and C (catechin). This result implied that two strains might hold a tannase. We considered the *H. camelliae* strain was an excellent source to investigate the potential in the production of tannases owing to its benefits to the host. Thus, this study aimed to identify, clone, express, purify a tannase from *H. camelliae* WT00C and investigate its enzymatic characteristics.

Herein, we have discovered the tannase coding gene from the genome of *H. camelliae* WT00C and successfully expressed the soluble protein in *E. coli* host cells. Furthermore_,_ the recombinant enzyme was purified to homogeneity through Ni-affinity chromatography, and its kinetic features have been thoroughly investigated.

## Results

### Putative tannase in *H. camelliae* WT00C

**Fig. 1:**
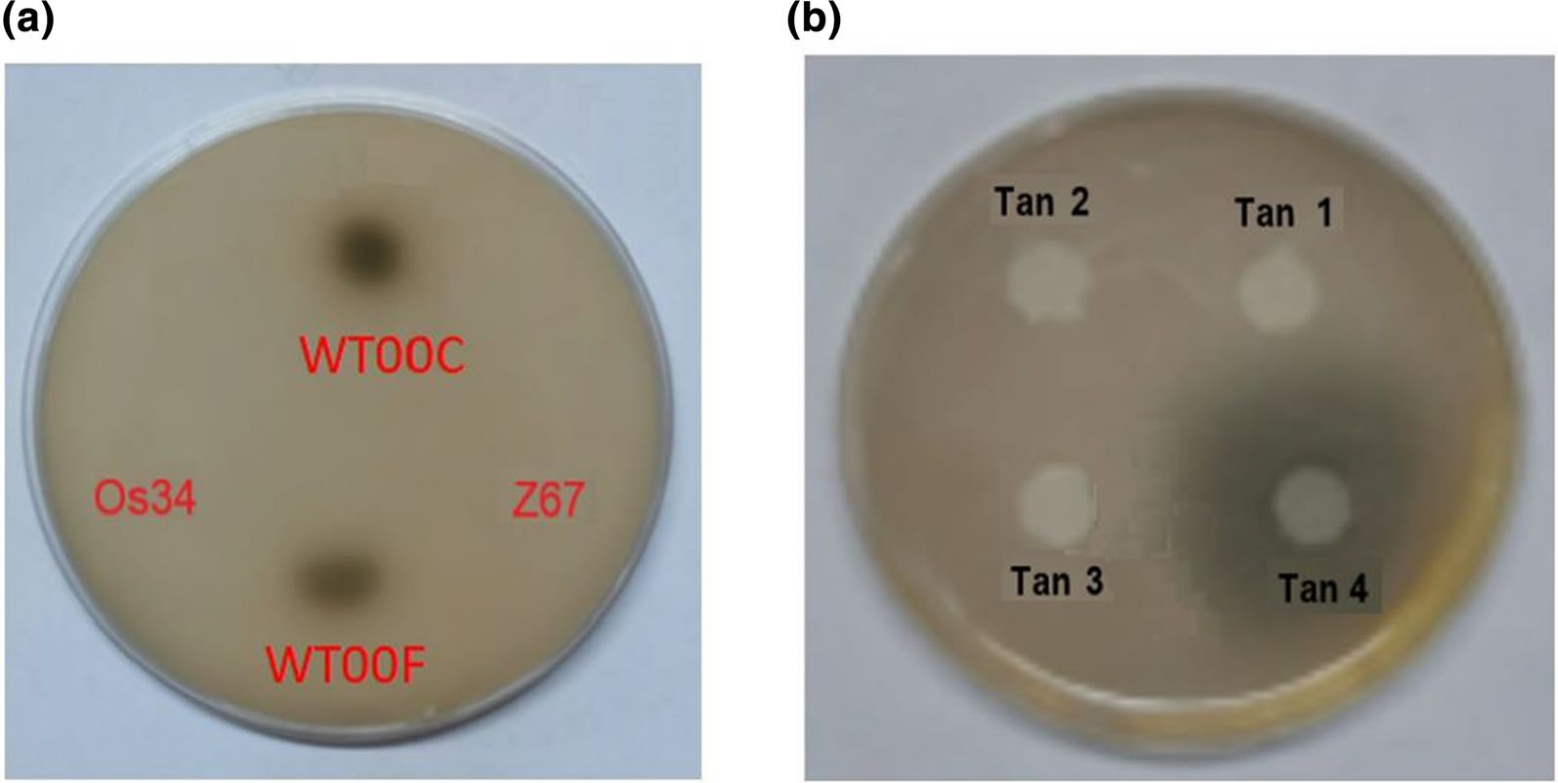
Plate assay of tannase activities. **a** Bacterial cells. WT00C: *H. camelliae* WT00C; WT00F: *H. camelliae* WT00F; Z67: *H. seropedica*e Z67; Os34: *H. rubrisubalbicans* Os34. **b** The purified enzymes. Tan1-4: four enzymes expressed respectively by 4 putative genes of *H. camelliae* WT00C in *E.coli*

Plate assay of bacterial tannases showed that *H. camelliae* WT00C and WT00F displayed tannase activity, whereas graminaceous endophytes *H. seropedica*e Z67 and *H. rubrisubalbicans* Os34 did not show any activity (Fig. 1a). This result suggested that *H. camelliae* WT00C and WT00F might have a tannase degrading tannic acid. Since no tannase gene was annotated in the genome of H. camelliae WT00C (Acc#: KV880769.1), we attempted to find those genes encoding the proteins containing the active site motif Gly-X-Ser-X-Gly. 12 ORFs encoding the polypeptides with the GXSXG motif were found in the genome of *H. camelliae* WT00C, in which four genes encoding the proteins with the mass of > 10 kDa were chosen for further study.

Multiple sequence alignment of the above four proteins and other seven microbial tannases from the database showed that only the 39 kDa protein encoded by the 4th gene shared two conserved motifs (GXSXG and DXXDXXD) with seven annotated tannases (see Additional file 1). In contrast, the other three proteins only hold a GXSXG motif. Thus, the result of the alignment indicated that the 39 kDa protein might be an active tannase.

### Gene cloning, tannase activity and phylogenetic tree

**Fig. 2:**
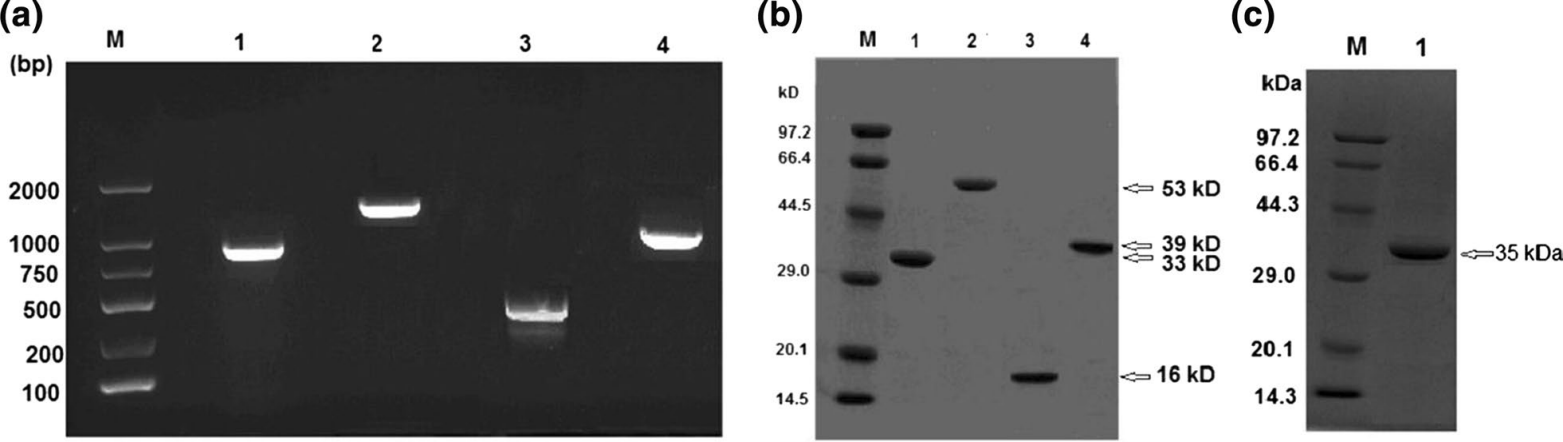
PCR products and the purified proteins. **a** 0.8% agarose gel showing DNA fragments amplified by PCR from 4 genes of *H. camelliae* WT00C. M: DNA marker; 1–4: PCR products of 4 genes; **b** 10% SDS-PAGE showing 4 proteins purified by Ni-affinity chromatography. M: protein standard; 1–4: the purified proteins expressed respectively by 4 genes; **c** 10% SDS-PAGE showing the truncated Tan_Hcw_. M: protein standard; 1: the truncated Tan_Hcw_

In order to identify which protein exhibits tannase activity, we attempt to amply the four putative tannase-encoding genes via PCR. PCR amplification gave the sizes of 900, 1458, 450 and 1107 bp for four different gene fragments, respectively (shown in Fig. 2a). After gene cloning, protein expression and purification, four proteins showed homogeneity on an SDS-PAGE gel, giving four resulting bands in each lane with the correct apparent molecular masses of 33, 53, 16 and 39 kDa, respectively (see Fig. 2b). Next, the activity of putative enzymes was measured using two different methods. The plate assay showed that only 39 kDa protein displayed tannase activity when applying tannic acid as substrate (Fig. 1b). Another method was a colorimetric assay that monitoring the absorbance increase caused by the release of the reaction product, gallic acid, at 520 nm [11]. The result was in agreement with the result of the plate assay, revealing that only the 39 kDa protein possesses tannase activities of 19.2 U/mg towards MG (methyl gallate) and 58.3 U/mg towards EGCG. Other 33, 53, and 16 kDa proteins did not show any detectable activity whether MG or EGCG was used as substrate. This consequence confirmed the speculation from the alignment of amino acid sequences. Hereinafter, the gene encoding the 39 kDa protein was defined as *tan*_Hcw,_ and its corresponding enzyme was named as Tan_Hcw._

**Fig. 3:**
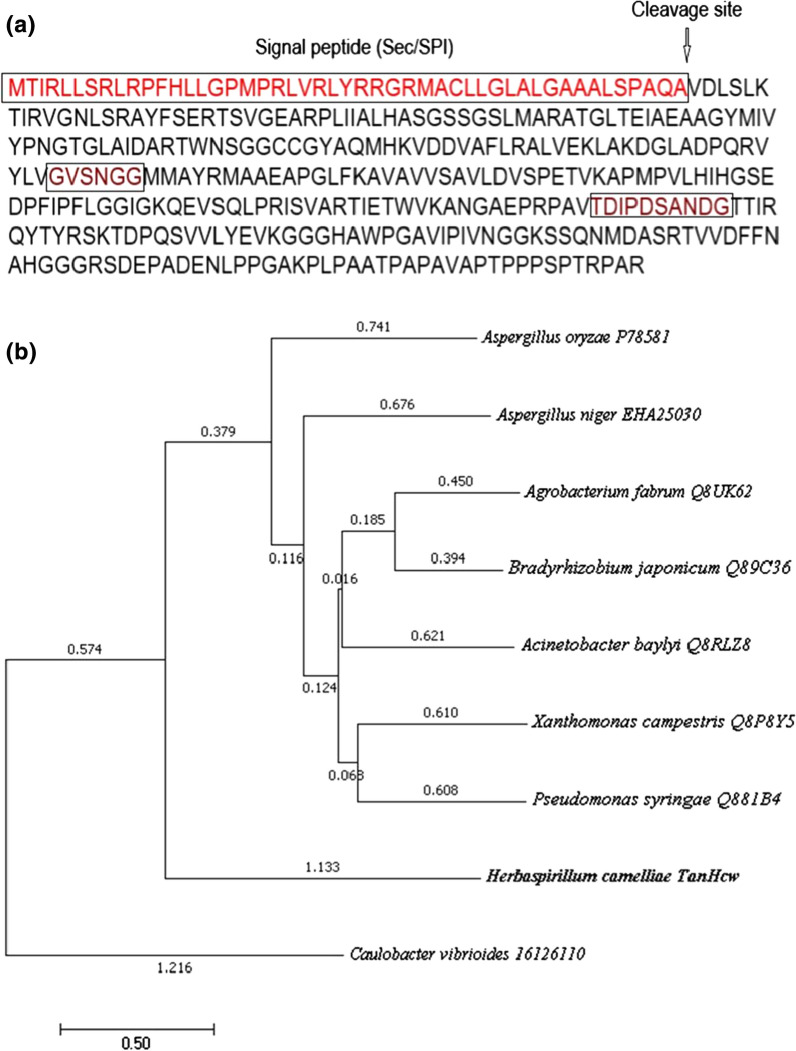
Amino acid sequence of Tan_Hcw_ and its phylogenetic tree. **a** Amino acid sequence of Tan_Hcw_. Prediction of the signal peptide by SignalP 5.0 program (http://www.cbs.dtu.dk/services/SignalP/). Signal peptide (Sec/SPI) was marked by an oblong box, and the cleavage site was also labeled with an arrowhead. Two conserved motifs (GXSXG and DXXDXXXD) were marked with a brown box. **b** The phylogenetic tree of Tan_Hcw_ and other tannases. Multiple sequence alignment was carried out using ClustalW (https://www.genome.jp/tools-bin/clustalw). The phylogenetic tree was constructed by using MEGA7.0 software (http://www.megasoftware.net/). The bar represents 0.5 amino acid substitutions per site

Furthermore, a phylogenetic tree was constructed to investigate the phylogenetic position of Tan_Hcw_. As shown in Fig. 3b, Tan_Hcw_ was located between *Caulobacter vibrioides* α/β-hydrolase and fungal and bacterial tannases and positioned in an independent branch. Its position in the tree exhibited a distant phylogenetic relationship with other bacterial tannases. From an evolutionary point of view, Tan_Hcw_ appeared to be closer to *Aspergillus* tannases rather than bacterial tannases. Since its phylogenetic relationship was distant from other bacterial tannases, Tan_Hcw_ could be a novel member of the tannase family based on its enzyme activity.

### Enzymatic characterization of Tan_Hcw_

The *tan*_*Hcw*_ gene, encoding a protein of 368 amino acids. ProtParam (https://web.expasy.org/protparam/) showed its molecular weight of 38,799.68, a theoretical pI of 9.44 and a grand average of hydropathicity of − 0.027. Prediction of the signal peptide by SignalP 5.0 program gave a Sec/SPI signal sequence (48 amino acids) at the N-terminus of Tan_Hcw_ (Fig. 3a), which suggested Tan_Hcw_ was a secretory protein that could be secreted from the cytoplasm to periplasm or extracellular medium. As shown in Fig. 3a, the cleavage site for the signal peptide was present between the position of 48 and 49 amino acid residues (QA-VD). We constructed a truncated *tan*_Hcw_ gene encoding the protein without 48 amino acids at N-terminus and expressed it successfully in *E. coli*. The truncated enzyme with a mass of 35 kDa was purified to homogeneity (shown in Fig. 2c). An active test showed that the truncated enzyme exhibited the same enzymatic activity as the untruncated Tan_Hcw_. In other words, removal of the signal peptide sequence did not affect the enzyme activity of Tan_Hcw._

**Fig. 4:**
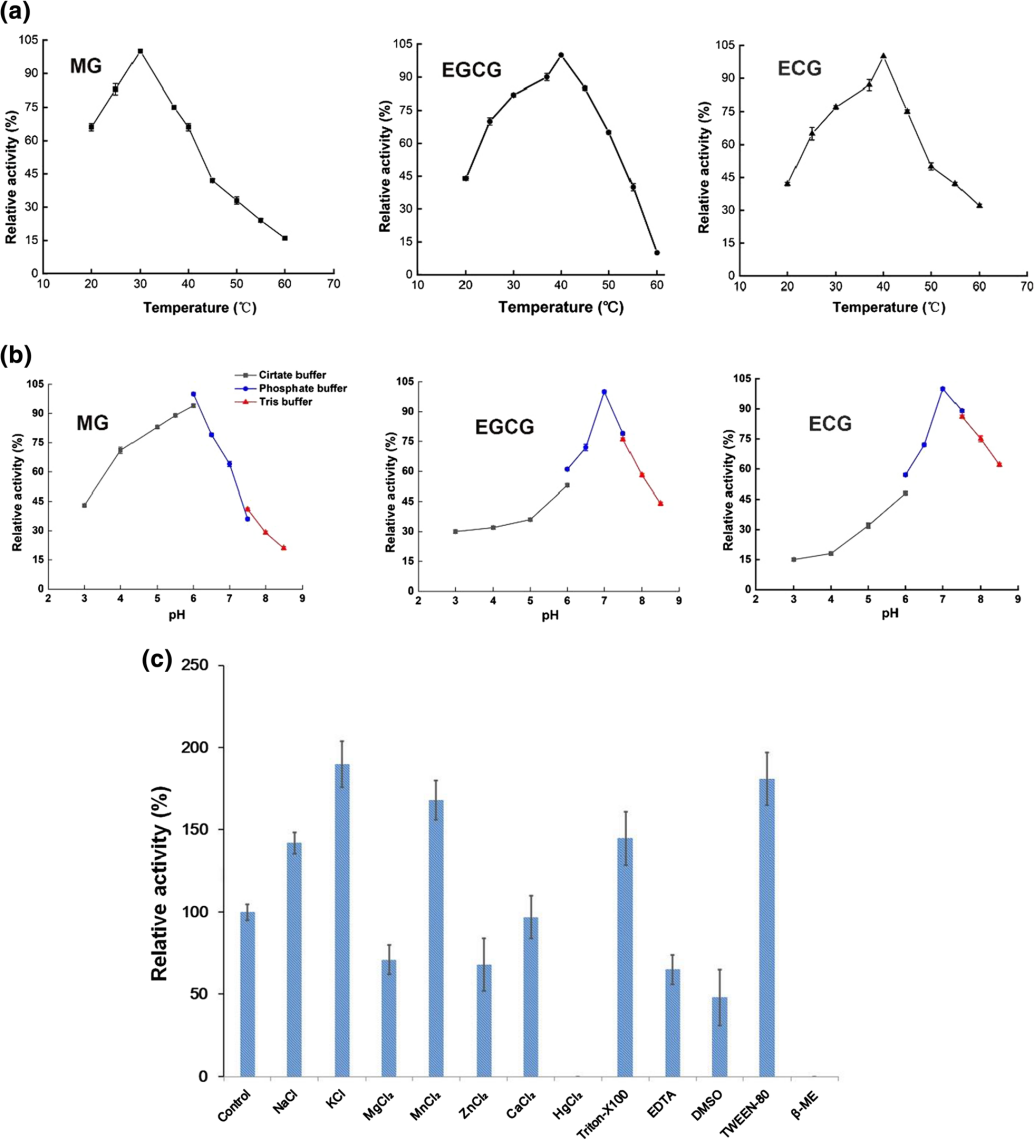
Enzymatic characterization of Tan_Hcw_. **a** Temperature effects on the enzyme activities of Tan_Hcw_ towards MG, EGCG and ECG; **b** pH effects on the enzyme activities of Tan_Hcw_ towards MG, EGCG and ECG; **c** Effects of metal ions, additive and organic solvents on the enzyme activities of Tan_Hcw_ towards EGCG. β-ME: β-mercaptoethanol

Tan_Hcw_ activity was assayed spectrophotometrically at a fixed concentration of MG (1 mM), EGCG (1 mM), and ECG (1 mM) over a range of temperatures and pH values. First, temperature-dependent was examined over a range from 20–60 °C. The results were shown in Fig. 4a, suggesting an optimal temperature of Tan_Hcw_ is 30 °C for MG and 40 °C for both EGCG and ECG. The optimal temperature was therefore adopted as the standard temperature for each assay. Then, pH-dependent was determined over the range from pH (3.0–8.5). Results from Fig. 4b revealed that the optimal pH of TanHcw is 6.0 for MG and 7.0 for both EGCG and ECG. Besides, the effects of metal ions, additives, organic solvents on the enzyme activities of Tan_Hcw_ were also evaluated under the optimal temperatures and pH values. The relative activity was measured in the assay solution supplemented with 1 mM Na^+^, K^+^, Ca^2+^, Zn^2+^, Mn^2+^, Mg^2+^, Hg^2+^, EDTA, or 1% EMSO, Triton-X100, Tween80, and β-mercaptoethanol. Figure 4c showed that Na^+^, K^+^ Mn^2+^, Triton-X100, and Tween80 increased the enzyme activity of Tan_Hcw_ from 42 to 90%, whereas Zn^2+^, Mg^2+^, Hg^2+^, EMSO, EDTA, and β-mercaptoethanol inhibited Tan_Hcw_ activity from 29 to 100%. Both Hg^2+^ and β-mercaptoethanol completely inhibited the enzyme activity of Tan_Hcw_. Among metal ions, only Ca^2+^ did not show noticeable activation or inhibition.

In addition, the detailed kinetic parameters of Tan_Hcw_ with MG, EGCG, and ECG have been evaluated under the standard conditions with the optimal temperatures and pH. Tan_Hcw_ showed typical Michaelis–Menten behavior at pH 7.0 and pH 6.0. Table 1 summarized all parameters obtained, and Additional file 1: Fig.S2 also exhibited Lineweaver–Burk plots of Tan_Hcw_ towards three substrates MG, EGCG, and ECG. Under pH 7.0 and 40 ºC, *K*_*m*_*, k*_*cat*_ and *k*_*cat*_* /K*_*m*_ of Tan_Hcw_ were 0.30 mM, 37.84 s^−1^, 130.67 mM^−1^ s^−1^ for EGCG and 0.33 mM, 34.59 s^−1^, 105.01 mM^−1^ s^−1^ for ECG. When MG was used as the substrate, *K*_*m*_*, k*_*cat*_, and *k*_*cat*_* /K*_*m*_ of Tan_Hcw_ were 0.82 mM, 14.64 s^−1^, 18.17 mM^−1^ s^−1^ at pH 6.0 and 30 °C. The data revealed that the catalytic efficiency (*k*_*cat*_* /K*_*m*_) of Tan_Hcw_ towards EGCG and ECG was tenfold larger than that towards MG. Analysis of catalytic efficiency implied that Tan_Hcw_ was more favorable to use EGCG and ECG as substrates.

**Table 1:** Kinetic parameters of the enzyme Tan_Hcw_ at the optimal temperatures and pH

Substrates	*K*_*m* (mM)_	*k*_*cat*__(s_^−1^_)_	*k*_*cat*_* /K*_*m* (mM_^−1^ s^−1^_)_	Assay conditions
EGCG	0.30 ± 0.01	37.84 ± 0.48	131	pH 7.0, 40 ºC
ECG	0.33 ± 0.01	34.59 ± 0.52	105	pH 7.0, 40 ºC
MG	0.82 ± 0.02	14.81 ± 0.31	18.17	pH 6.0, 30 ºC

### Molecular structure simulation of Tan_Hcw_

**Fig. 5:**
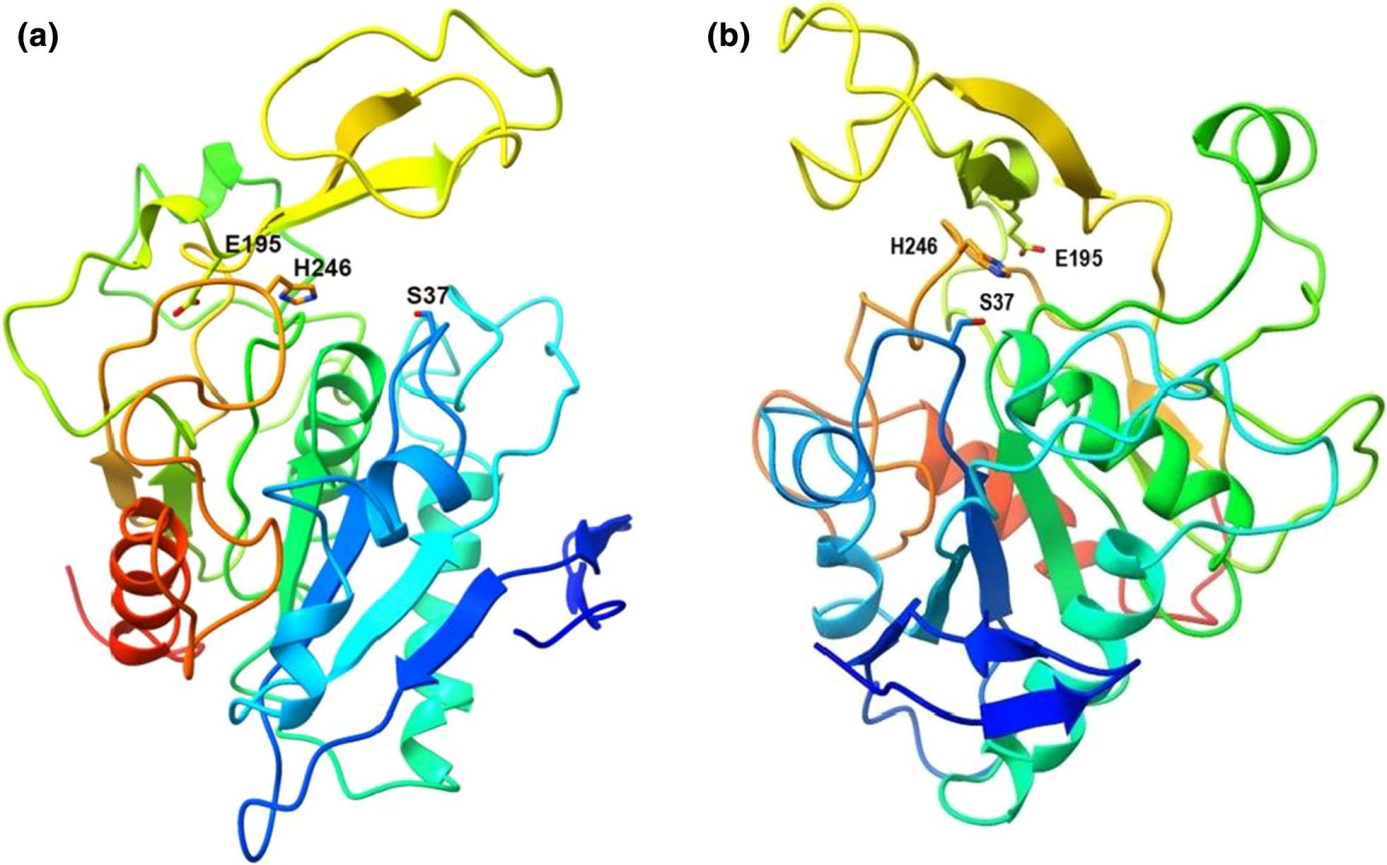
The predicted structure of *H. camelliae* Tan_Hcw._ **a** The overall structure of a monomer without a Sec/SPI signal peptide of 48 amino acids at the N-terminus. The side chains of three residues in the catalytic triad were shown in stick representation. **b** Another view of the monomer structure with a horizontal rotation of about 90° relative to **a**. The Swiss-model server (http://swissmodel.expasy.org/) was employed for homology modeling and the PyMOL program (http://pymol.sourceforge.net/) was used for structural analysis and figure production

The 3-D structure of Tan_Hcw_ protein was simulated by homology modeling using the crystal structure of the oxidized polyvinyl alcohol hydrolase (PDB ID: 3W16, the identity of 19.3%) as a template. The predicted structure of Tan_Hcw_ monomer with a signal peptide truncated at N-terminus was shown in Fig. 5. The overall structure of Tan_Hcw_ in the model displayed a typical α/β hydrolase fold composed of seven mixed β-strands flanked by five α-helixes and a flexible cap at the top of the active pocket. As compared to the structures of tannases reported previously in *Lactobacillus plantarum* (PDB ID:3WA6) [12, 13] and *Aspergillus oryzae* RIB40 (PDB ID: 3WMT) [14], Tan_Hcw_ shared not only similar α/β hydrolase fold but also comparable active pocket. Especially, its flexible cap on the active pocket was more similar to *L. plantarum* tannase. The common nucleophile-histidine-acid catalytic triad of α/β hydrolase fold proteins [15] was also identified in this model. In the active pocket, three amino-acid residues, Ser37, Glu195 and His246, were possibly involved in the catalytic activity of Tan_Hcw_ (Fig. 5). Interestingly, three catalytic residues of Tan_Hcw_ were not in its two conserved motifs (GXSXG and DXXDXXD).

## Discussion

Tannases have been employed to hydrolyze the ester and depside bonds of the tannic acid, releasing the gallic acid and glucose. In our previous study, we observed *H. camelliae* strain degraded EGCG and ECG to release GA and proposed it might have a tannase to catalyze this reaction. However, when searching the genome of *H. camelliae* WT00C, we could not find a tannase annotated in the NCBI database (KV880769.1). Based on the common active site motif (GXSXG) in the conserved domain of tannase (pfam07519), four genes encoding the proteins with the size of > 10 kDa were chosen for the initial analysis. Alignment of amino acid sequences showed that the 39 kDa protein encoded by the *tan*_Hcw_ gene harbors two conserved motifs GXSXG and DXXDXXD, while the other three proteins shared with one active site motif (GXSXG). Activity measurement of putative enzymes revealed that only the 39 kDa protein, annotated as prolyl oligopeptidase in the NCBI database, displayed tannase activity. Prolyl oligopeptidase is a cytosolic serine peptidase, hydrolyzing the proline-containing peptides at the carboxy terminus [16]. The protein of 39 kDa should not be a prolyl oligopeptidase because it is a secretary enzyme with a Sec/SPI signal peptide of 48 amino acids at the N-terminus. The detailed kinetic assay showed that the 39 kDa enzyme effectively catalyzes the degradation of MG, tannin, EGCG, and ECG. Taken together, these results suggest that the 39 kDa protein named Tan_Hcw_ is a newly discovered tannase belonging to the bacterial tannase family. Surprisingly, its position in the phylogenetic tree exhibited a distant relationship with other bacterial tannases, appearing closer to *Aspergillus* tannases,which indicates that Tan_Hcw_ could be a novel member of the tannase family. The structural simulation of Tan_Hcw_ suggests that this enzyme displayed a typical α/β hydrolase fold that is quite similar to other tannases in molecular structure. The catalytic triad of the α/β hydrolase fold was also identified in the predicted structure of Tan_Hcw_. Notably, its catalytic residue Ser37 is neither in the common active site motif (GXSXG) nor in the conserved motif DXXDXXD. Such phenomenon that enzyme structures are more conserved than sequences has also been observed in fungal tannases [17].

Substrate specificities of tannases from different organisms are quite different [18]. The gallotannin-decomposing tannases include two separate enzymes, an esterase and a depsidase, with specificities for methyl gallate and m-digallic acid ester linkages, respectively [19]. The tannase of *Lactobacillus plantorum* displays a higher esterase activity, while the tannase from *Streptomyces sviceus* exhibits a higher depsidase activity [18]. Tan_Hcw_ from *H. camelliae* hydrolyzes EGCG and ECG much effectively than MG, which suggests that Tan_Hcw_ may favorable to be a depsidase. Substrate specificity and activity of an enzyme may be related to the living environments of biological species. As a specific tea-plant endophyte, *H. camelliae* has evolved a novel tannase degrading EGCG and ECG effectively.

*H. camelliae* was isolated from ornamental tea plants (*Camellia sinensis*. L) [5]. When irrigation, sprinkling and traumatic infection were applied to infect different plants (e.g., *Brassica campestris*, *Brassica rapa*, *Oryza sativa*, *Triticum aestivum* and *Camellia sinensis*), *H. camelliae* went into plants via plant vulnus and only colonized in tea plant [7]. Colonization only in theaceous tea plants suggested that the host specificity of *H. camelliae* was quite specific as a specialist. In tea plants, tea polyphenols (TP) are the main active compounds in tea. TP is composed of catechins, flavonols, anthocyan, depsides and polymeric phenols, in which catechins are 65–80% of total TP. Catechins include epigallocatechin gallate (EGCG), epigallocatechin (EGC), epicatechin gallate (ECG), epicatechin (EC) and catechin (C) [20]. EGCG, as the most biologically active compound, is 65% of the total catechins in green tea [21, 22]. Besides antioxidative, anti-inflammatory and anti-carcinogenic activities, catechins (EGCG in particular) have also shown antimicrobial effects [23–26]. Our recent study examined the biological effects of catechins on three plant-endophytic *H. camelliae* WT00C, *H. seropedicae* Z67 and *H.rubrisubalbicans* Os34 (unpublished data). The latter two graminaceous endophytic bacteria were used as reference. It has been found that only *H. camelliae* grew in fresh tea juice and displayed strong tolerance to catechin compounds. Moreover, it suggested that catechin compounds in tea plants, EGCG, EGC and ECG in particular, played a critical role in limiting bacterial colonization in tea plants. As a specific endophyte in tea plants, *H. camelliae* must defend the antimicrobial effects of free catechins in the tea plant for its survival. Once *H. camelliae* enters the tea plant, it secretes Tan_Hcw_ and then degrades EGCG and ECG effectively. The decrease of active compounds in the tea plant benefits bacterial colonization and growth. Therefore, it is reasonable to assume that Tan_Hcw_ has a crucial role in the specific colonization of *H. camelliae* in tea plants. Discovery of the tannase Tan_Hcw_ in this study may give us a reasonable explanation for specific colonization and vigorous growth of *H. camelliae* in tea plants.

To date, the filamentous fungi *Aspergillus* species are the primary source to produce commercially available tannases. Tan_Hcw_ exhibited relatively high affinities for MG among the reported filamentous fungi tannases (Additional file 1: Table S1). The affinity of Tan_Hcw_ to MG (*K*_*m*_ value:0.82 mM) was similar to the enzyme from *A. oryzae* (1.11 mM) [27] but lower than that observed for the enzyme produced by from *Arxula adeninivoran* (3.5 mM) [28]*, Aspergillus fumigatus* (6.3 mM) [29]*, and Aspergillus niger* (5.2 mM) [30]. The catalytic efficiencies (*k*_*cat*_ / *K*_*m*_) of Tan_Hcw_ for EGCG and ECG were 130 mM^−1^ s^−1^ and 105 mM^−1^ s^−1^, which are the 2nd highest *k*_*cat*_ / *K*_*m*_ values for EGCG and ECG of all known highly efficient tannases in the literature. The highest catalytic efficiencies towards EGCG (260.76 mM^−1^ s^−1^) and ECG (195.3 mM^−1^ s^−1^) were determined with the tannase from *L. paraplantarum* [31]. Notably, the kinetic parameters may not be comparable different when different assay methods and conditions were applied. As mentioned above, green tea contains high amounts of EGCG, which is related to astringency and bitterness. Thus, Tan_Hcw_ might be applicable in the industrial production of tea beverages to improve the quality of green tea extracts.

## Conclusions

In this study, a novel enzyme Tan_Hcw_ displaying tannase activity was identified in the tea-plant endophyte *Herbaspirillum camelliae*, and its enzyme characterizations were investigated. Tan_Hcw_ is a secretary enzyme with the Sec/SPI signal peptide of 48 amino acids at the N-terminus. Effective hydrolysis of EGCG and ECG by Tan_Hcw_ decreases the concentration of free catechins in the tea plant. As *H. camelliae* is an endophytic bacterium colonizing specifically in tea plants, degradation of the main active compounds benefits bacterial colonization and growth in tea plants. Thus, the tannase activity of Tan_Hcw_ may play a crucial role in determining the host specificity of *H. camelliae* as a specialist in tea plants. Investigating the detailed kinetic characteristics of Tan_Hcw_ advances our knowledge about the bacterial tannase at the molecular level and paves the way for the large-scale application of bacterial tannase.

## Methods

### Bacterial strains and chemicals

*H. camelliae* WT00C and WT00F were isolated from the tea plant in Wuhan City, China [5] and stored in our laboratory. *H. rubrisubalbicans* Os34 [32] was given by Zhejiang University as a gift, and *H. seropedica*e Z67 [33] (#ATCC35892) was purchased from ATCC (American Type Culture and Collection). Other bacterial strains (e.g., *E. coli* DH5α and BL21(ED3) *pLys*S) used in this study were stored in our laboratory. These bacterial strains were usually cultured in LB medium at 37℃. Methyl gallate (MG), tannic acid, gallic acid (GA), (–)–epicatechin-3-gallate (ECG), and (–)–epigallocatechin-3-gallate (EGCG) were purchased from Sigma-Aldrich, USA. Inorganic and organic reagents, as well as culture media, were purchased from Zhong Ke (Shanghai, China).

### Gene cloning, protein expression and purification

The primers for the amplification of 4 genes were designed and synthesized (see Table 2). PCR was performed by using *TransTaq*® HiFi DNA polymerase (TransGen Biotech, Beijing, China), the primer pairs listed in Table 2, and the genomic DNA of *H. camelliae* WT00C as a template. PCR was initiated by preheating the reaction mixture at 95 ºC for 5 min, followed by 30 cycles of denaturing at 95 ºC for 30 s, annealing at 56 ºC for 1 min and extension at 72 ºC for 1.5 min with a final extension at 72 ºC for 5 min. The DNA fragments amplified by PCR were recovered using Zymoclean™ Gel DNA Recovery Kit (Yanxin Biotechnology Co., Ltd, Guangzhou, China) and respectively inserted into a pMD18-T plasmid. The recombinant pMD18-T plasmid was transformed into *E.coli* DH5α for DNA amplification in vivo. Eventually, the recombinant pMD18-T plasmid was extracted, and each gene was sequenced by BGI (Beijing Genomics institution).

**Table 2:** Information about 4 genes cloned from the genome of *H. camelliae* WT00C and the primers used in PCR

Gene	Primers	DNA sequence of the primer	Gene size (bp)	Gene position at the genomic DNA	Protein name annotated in NCBI database
1	Tan1-F Tan1-R	5’-GGAATTCCATATGACGTCTTCTTTCATCTGGA-3’ 5’-CCGCTCGAGGCCCAGCAGGAACTGG-3’	900	3778213–3779112	alpha/beta hydrolase
2	Tan2-F Tan2-R	5’-GGAATTCCATATGACGAAGCTACCCGATAAC-3’ 5’-CCGCTCGAGCGCCCCCTTGTAAGGG-3’	1458	3719942–3721399	GGDEF,domain-containing protein
3	Tan3-F Tan3-R	5’-GGAATTCCATATGCAGGGATCGGCG-3’ 5’-CCGCTCGAGTTCGGCCAGGCTGAC-3’	450	5544953–5545402	acyl-CoA dehydrogenase family protein
4	Tan4-F Tan4-R	5’-GGAATTCCATATGGTCGATCTGTC-3’ 5’-CCGCTCGAGACGTGCCGGACGGGT-3’	1107	4574529–4575635	prolyl oligopeptidase family serine peptidase/ alpha/beta hydrolase

4 genes confirmed by DNA sequencing were respectively inserted into the pET23a plasmid and then transformed into *E. coli* BL21(DE3) *pLys*S. Positive transformants were selected on LB plates containing 100 μg/ml ampicillin, and confirmed by PCR. A single colony was inoculated into 5 ml LB broth plus 100 μg/ml ampicillin and incubated at 37 °C overnight. The bacterial culture was then inoculated with a ratio of 1:100 into 500 ml fresh LB broth containing 100 μg/ml ampicillin and grew at 25 °C, 200 rpm. When OD_600_ of the culture approached 0.6, protein expression was initiated by adding IPTG to the final concentration of 0.5 mM. After IPTG inducement at 25 °C for 4 h, the bacterial cells were harvested by centrifuging at 4 °C, 6000 rpm for 15 min. The pellets of bacterial cells were re-suspended in 50 mM Tris–HCl (pH 8.0) and broken by sonication. The crude extracts were collected and clarified by centrifuging at 4 °C, 12,000 rpm for 15 min. As each protein carried with a His-tag at its C-terminus, Ni-affinity chromatography was thus used to purify the protein. Ni-affinity column was equilibrated with 50 mM Tris–HCl (pH 8.0), washed with 10 mM imidazole in 50 mM Tris–HCl (pH 8.0), and eluted gradient with 10–200 mM imidazole in 50 mM Tris–HCl (pH 8.0). Protein purity was routinely monitored on 10% SDS-PAGE, and protein concentration was estimated by measuring the absorbance at the wavelength of 280 nm and calculated by using its extinction coefficient.

### Identification of tannase activity

Tannase activities of *H. camelliae* WT00C and WT00F were tested according to the method reported by Kumar et al. [34]. In the assay, *H. seropedica*e Z67 and *H. rubrisubalbicans* Os34 were used as the negative control. Initially, four strains were respectively cultured in LB medium at 37 °C, 200 rpm until OD_600_ of 0.8. Bacterial cells were then collected and dropped separately on the surface of nutrient agar plates containing 2% tannic acid. The plates were incubated at 37 °C for 3 days. During the incubation of plates, tannic acid added in the nutrient agar interacted with proteins to form a tannin-protein complex, and then the tannin-protein complex was cleaved by bacterial tannases to form a greenish brown zone around bacterial colonies in the plate [12]. Finally, the result was recorded by photography.

Tannase activities of 4 proteins expressed by 4 cloned genes were tested using two methods. One was the plate assay described above. 4 purified proteins (50 μg for each) were respectively dropped on the surface of the nutrient agar plate containing 2% tannic acid, and the plate dish was then incubated at 37 °C for 12 h. The greenish-brown zone was recorded by photography. Another method by measuring A_520_ of the chromogen formed between gallic acid (GA) and rhodamine was also employed [34]. In the assay, 0.25 mL of 10 mM MG dissolved in 50 mM citrate buffer (pH 5.0) were added to the blank and test tubes. All tubes were incubated at 30 °C for 5 min. Then, 0.25 mL of 50 mM citrate buffer (pH 5.0) and 0.25 mL of the enzyme sample were respectively added to the blank and test tubes, and the reaction mixtures were kept at 30 °C for 5 min. 0.3 mL of 0.667% rhodanine dissolved in 100% methanol (w/v) was added to all the tubes, and the tubes were kept at 30 °C for 5 min. 0.2 mL of 0.5 M KOH was then added to each tube and incubated at 30 °C for 5 min. Finally, each tube was diluted with 4.0 mL dH_2_O and incubated at 30 °C for 10 min and the absorbance was recorded against water at 520 nm on a Shimadzu UV/visible spectrophotometer (UV-2550). The enzyme activity was calculated from the change in absorbance: ΔA_520_ = (A_test_—A_blank_). Meanwhile, the standard curve for GA concentrations was also obtained by measuring A_520_ based on chromogen formation between GA and rhodamine.

### Enzymatic assays

Based on the protocol reported by Sharma et al. [11] and Tomas-Cortazar et al. (2018) [35], the activities of Tan_Hcw_ from *H. camelliae* WT00C were measured at pH 5.0 over a range of temperatures (20–60 °C) to determine the optimal temperature of enzyme activity. The optimal pH was also determined by measuring enzyme activities at different pH (3.0–8.5) under the optimal temperatures. The pH buffers were 50 mM citrate buffer for pH 3.0–6.0, 50 mM phosphate buffer for pH 6.0–7.5, and 50 mM Tris–HCl for pH 7.5–8.5. To test the effects of metal ions and other reagents on enzyme activity, 1 mM of KCl, CaCl_2_, MaCl_2_, MnCl_2_, HgCl_2_, ZnCl_2_, EDTA, and 1% Triton 80, DMSO, β-mercaptoethanol were respectively added to the reaction mixture. In the assay, MG, EGCG, or ECG was respectively used as the substrate, and the final concentration of each substrate was 1 mM. 10 μg of Tan_Hcw_ enzyme was added in each test tube. In blank tubes, the buffer was used instead of the enzyme sample, and other reaction reagents were the same as those in test tubes. Enzyme reactions were performed as described above. Finally, each tube was diluted with 4.0 mL dH_2_O and incubated at 30 °C for 10 min, and the absorbance was recorded against water at 520 nm on a Shimadzu UV/visible spectrophotometer (UV-2550). Enzyme activity (U/ml) was defined as units of activity per milliliter of enzyme solution, where one unit (U) represented 1 μmol of gallic acid formed per minute. Specific activity (U/mg) was expressed as units of activity per milligram of the enzyme. All measurements were performed in triplicate and error bars represent sample standard deviation.

Kinetic parameters of Tan_Hcw_ enzyme for MG, EGCG, and ECG were monitored by varying substrate concentrations. The enzyme reactions were performed at 30 °C, pH 6.0 for MG, and 40 °C, pH 7.0 for EGCG and ECG. Rates were measured spectrophotometrically at the wavelength of 520 nm over a range of substrate concentrations (0.1–2.0 mM). Michaelis–Menten parameters were calculated using the UVProbe-[Kinetics] version 1.11a (SHIMADZU Corporation), and kinetic parameters were determined by Lineweaver–Burk plot [36] and checked by Hanes-Woolf and Eddie-Hofstee plots. The deviation between the same parameters obtained from different plots was less than 5%. In each plot, the correlation coefficient (r^2^) value was equal to or large than 0.997. All data were also analyzed using the statistic software SPSS based on the non-linear regression method INVERSE, and analysis of variance gave P values of less than 0.005 in each case.

## Supplementary Information

**Additional file 1: Fig. S1**. Alignment of amino acid sequences of microbial tannases and 4 putative tannases in H. camelliae WT00C. * pfam07519: a conserved domain of tannases predicted by NCBI database (https://www.ncbi.nim,nih.gov/conserved domain/tannase); Acinetobacter: A. bayli (Q8RLZ8); Xanthomonas: X. campestris (Q8P8Y5); Agrobacterium: A. fabrurn (Q8UK62); Bradyrhtzobium: B. japonicum (Q89C36); Pseudomonas: P. syringae (Q88IB4); Aspergillus: A. niger (EHA25030); Tan1-4: putative enzymes of Herbaspirillum camelliae.** Fig. S2**. Kinetic parameter determination of TanHcw at the optimal temperatures and pH. (a) Lineweaver-Burk plot for substrates EGCG at pH7.0 and 40 ºC; (b) Lineweaver-Burk plot for substrates ECG at pH7.0 and 40 ºC; (c) Lineweaver-Burk plot for substrates MG at pH6.0 and 30 ºC.** Table S1**. Kinetic parameters of TanHcw compared with other tannases.

## Data Availability

All data and material have included in this paper.
