# Supplementary material for: Degradation of epigallocatechin and epicatechin gallates by a novel tannase TanHcw from Herbaspirillum camelliae
Source: Microb Cell Fact. 2021 Oct 12;20:197. doi: 10.1186/s12934-021-01685-1 (PMC8507159; doi:10.1186/s12934-021-01685-1)
Supplement: Supplementary file 1 — Additional file 1: Fig. S1. Alignment of amino acid sequences of microbial tannases and 4 putative tannases in H. camelliae WT00C. * pfam07519: a conserved domain of tannases predicted by NCBI database (https://www.ncbi.nim,nih.gov/conserved domain/tannase); Acinetobacter: A. bayli (Q8RLZ8); Xanthomonas: X. campestris (Q8P8Y5); Agrobacterium: A. fabrurn (Q8UK62); Bradyrhtzobium: B. japonicum (Q89C36); Pseudomonas: P. syringae (Q88IB4); Aspergillus: A. niger (EHA25030); Tan1-4: putative enzymes of Herbaspirillum camelliae. Fig. S2. Kinetic parameter determination of TanHcw at the optimal temperatures and pH. (a) Lineweaver-Burk plot for substrates EGCG at pH7.0 and 40 ºC; (b) Lineweaver-Burk plot for substrates ECG at pH7.0 and 40 ºC; (c) Lineweaver-Burk plot for substrates MG at pH6.0 and 30 ºC. Table S1. Kinetic parameters of TanHcw compared with other tannases. [file 12934_2021_1685_MOESM1_ESM.docx]

**Supplementary Figure**


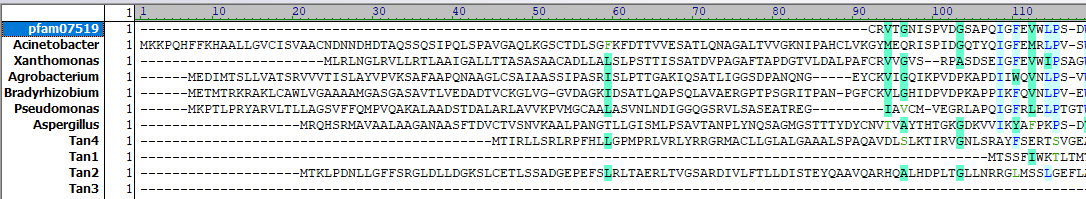


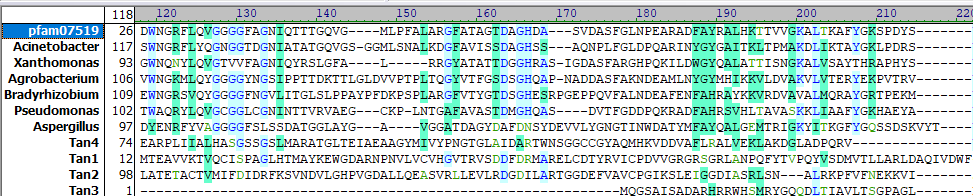


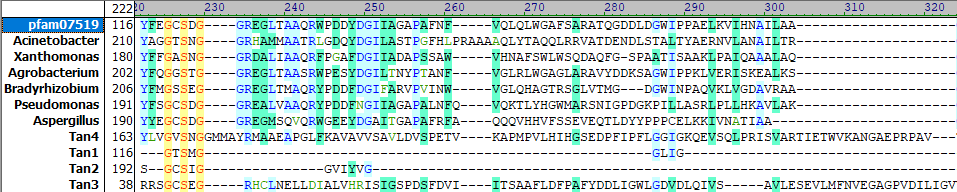


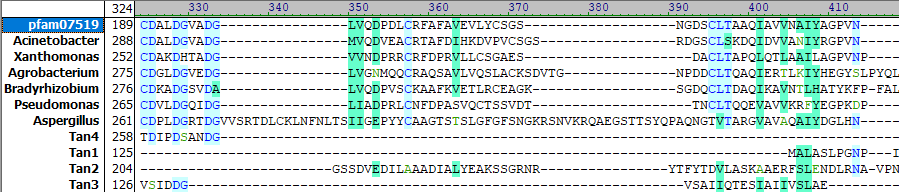


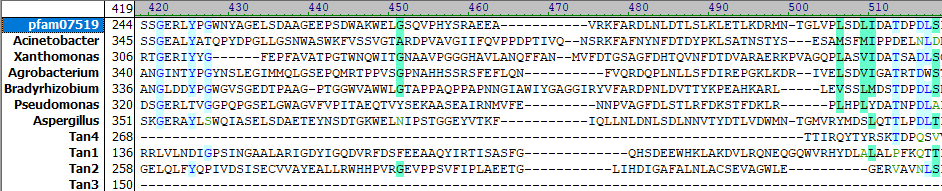


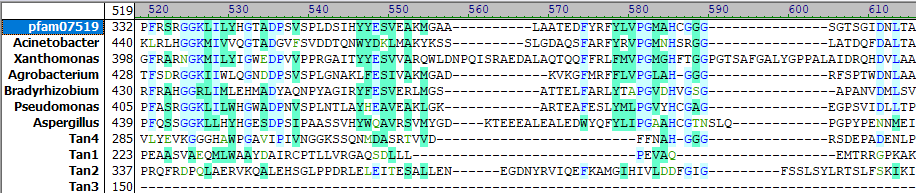


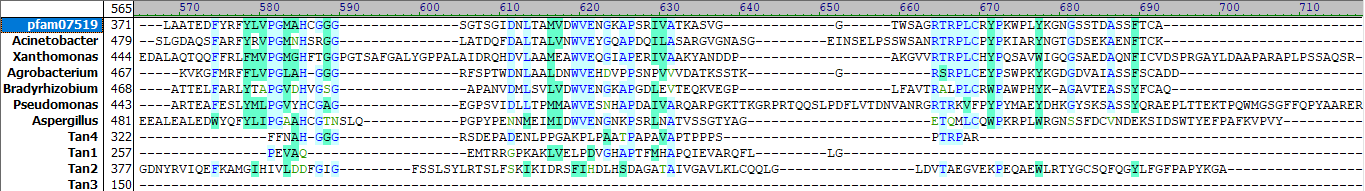


**Fig**.S**1.** Alignment of amino acid sequences of microbial tannases and 4 putative tannases in *H.* camelliae WT00C

* pfam07519: a conserved domain of tannases predicted by NCBI database (<https://www.ncbi.nim,nih.gov/conserved> domain/tannase); Acinetobacter: *A. bayli* (Q8RLZ8); Xanthomonas: *X. campestris* (Q8P8Y5); Agrobacterium: *A. fabrurn* (Q8UK62); Bradyrhtzobium: *B. japonicum* (Q89C36); Pseudomonas: *P. syringae* (Q88IB4); Aspergillus: *A. niger* (EHA25030); Tan1-4: putative enzymes of *Herbaspirillum* camelliae


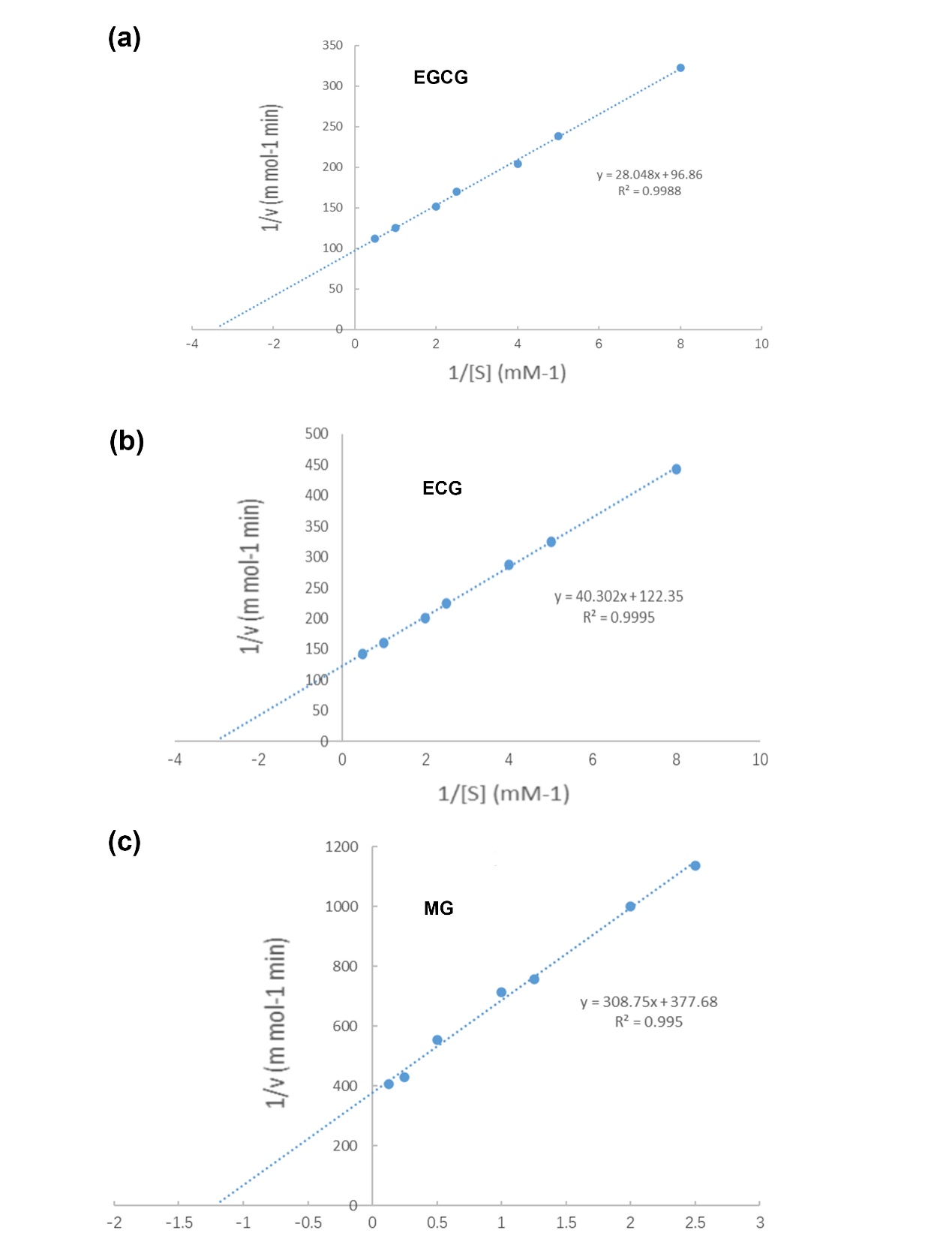


**Fig**.S2 Kinetic parameter determination of Tan_Hcw_ at the optimal temperatures and pH. (a) Lineweaver-Burk plot for substrates EGCG at pH7.0 and 40ºC; (b) Lineweaver-Burk plot for substrates ECG at pH7.0 and 40ºC; (c) Lineweaver-Burk plot for substrates MG at pH6.0 and 30ºC.

**Table S1**. Kinetic parameters of Tan_Hcw_ compared with other tannases.

| Organism | Substrate | Kinetic parameters | | | Assay  conditions | Reference |
| --- | --- | --- | --- | --- | --- | --- |
|  |  | *K_m_* _(mM)_ | *k_cat_* _(s_^-1^_)_ | *k_cat_ /K_m_* _(mM_^-1^ _s_^-1^_)_ |  |  |
| *Lactobacillus paraplantarum* | EGCG  ECG  MG | 0.06  0.06  0.5 | 14.3  11.1  72.7 | 260.7  195.3  145.1 | pH 8.0, 37°C | [31] |
| *Lactobacillus plantarum* | EGCG  ECG  MG | 0.1  0.03  0.37 | 1.12  1.49  46 | 11.7  52.2  125 | pH 8.0, 37°C | [31] |
| *Lactobacillus pentosus* | EGCG  ECG  MG | 0.06  0.05  0.87 | 0.44  0.42  16 | 7.25  8.63  18.8 | pH 8.0, 37°C | [31] |
| *Aspergillus oryzae* | EGCG  ECG  MG | 0.24  0.36  1.11 | 7.0  9.3  27.7 | 29.9  25.8  25 | pH 6.0, 30℃ | [27] |
| *Arxula adeninivoran* | MG | 3.5 | 0.39 | 0.11 | pH 6.0, 30°C | [28] |
| *Aspergillus fumigatus* | MG | 6.28 | 152.86 | 24.3 | pH 5.0, 30°C | [29] |
| *Aspergillus niger* | MG | 5.17 | 78.01 | 15.1 | pH 5.0, 30°C | [30] |
| *Herbaspirillum camelliae* | EGCG  ECG  MG | 0.3  0.33  0.82 | 37.8  34.6  14.8 | 131  105  18.2 | pH 7.0, 40ºC  pH 7.0, 40ºC  pH 6.0, 30ºC | this study |
